# Fruitful exchanges: social networks and food resources amidst change

**DOI:** 10.1186/s40066-021-00342-5

**Published:** 2022-02-16

**Authors:** Sam Schramski, Ana Carolina Barbosa de Lima

**Affiliations:** 1 Center for the Analysis of Social-Ecological Landscapes, Indiana University, Bloomington, IN 47405 USA; 2 Project ECHO, University of New Mexico, Albuquerque, NM 87102 USA

**Keywords:** Agrobiodiversity, Social network analysis, Food exchanges, Amazonia, Riverine peoples, Sustainable development

## Abstract

**Background:**

The Amazon region of Brazil is known both for its significant biological and cultural diversity. It is also a region, like many parts of the country, marked by food insecurity, even amongst its rural agricultural populations. In a novel approach, this paper addresses the networks of exchanges of local food and their relationship to the agrobiodiversity of traditional riverine peoples’ (*ribeirinho*) households in the Central Amazon. Methodologically, it involves mapping the social networks and affinities between households, inventories of known species, and, finally, statistical tests of the relationships between network and subsequent agrobiodiversity.

**Results:**

The diversity per area of each land type where food cultivation or management takes place shows how home gardens, fields and orchards are areas of higher diversity and intense cultivation compared to fallow areas. Our findings, however, indicate that a household’s income does appear to be strongly associated with the total agrobiodiversity across cultivation areas. In addition, a household’s agrobiodiversity is significantly associated with the frequency and intensity of food exchanges between households.

**Conclusions:**

Agrobiodiversity cannot be considered separate from the breadth of activities focused on sustenance and yields from the cash economy, which riverine people engage in daily. It seems to be connected to quotidian social interactions and exchanges in both predictable and occasionally subtler ways. Those brokers who serve as prominent actors in rural communities may not always be the most productive or in possession of the largest landholdings, although in some cases they are. Their proclivity for cultivating and harvesting a wide diversity of produce may be equally important if not more so.

**Supplementary Information:**

The online version contains supplementary material available at 10.1186/s40066-021-00342-5.

## Background

The COVID-19 pandemic has shown a light on the preexisting concern with food security globally [1–4], as researchers contend that the pandemic period could render the many vulnerable households insecure for long after the SARS-CoV-2 virus is largely contained. Already between 2015 and 2019, food insecurity rose throughout the world, despite successes in fighting hunger in years prior [5]. While the majority of the world’s population lives in urban geographies [6], and the 2020 FAO report and others emphasize the food insecure plight of urban denizens, statistically, rural households are still more likely to be food insecure [7]. Outside of the continuous presence of the COVID-19 pandemic, the leading culprits for food insecurity on a global scale have been the effects of climate change on agriculture and the difficulty of access to nutritious foods [5].

Brazil find itself in a similarly pressing situation, as a recent report on food insecurity shows more than 40% of the rural population is food insecure, with the North (Amazon) and Northeast regions being the most affected [8]. From an economic, social, and cultural perspective, the complexity of understanding the causes and consequences of food security in the Brazilian Amazon has proved to be fertile ground for research [9–11]. Much of the cited work takes place in riverine communities in which households practice a mix of agriculture and fishing livelihoods, but it would not be inaccurate to argue that most of these rural communities are at least nominally agricultural.

From a food studies perspective, most studies evaluating rural and riverine Amazonian local diets portray them as monotonous and lacking in variety [12, 13]. Swidden agriculture and agroforestry are particularly central to diets in the Middle Solimões region, where manioc (*Manihot esculenta*), and to a lesser degree açaí (*Euterpe oleracea*), provide substantial calories [14]. While the price of agricultural goods has fluctuated in the last several years—particularly for manioc flour, a chief staple [15]—they are still periodically delivered to local and regional markets [16]. Of note, a diverse array of locally produced crops may increase the desirability of consuming staples, such as fish and manioc flour, particularly if there is a limitation on adding variety through purchased foods.

Food variety and networks of food exchange, meanwhile, may be increasingly important for food security in the face of the effects of climate change throughout the Amazon, principally in terms of the intensification of droughts and floods in the Middle Solimões region [17]. Literature on the importance of exchanges of food crops in rural communities as one means of promoting food security amidst change has expanded as the ease in use of tools combining social network analysis (SNA) and agronomic and ecological methods becomes more widely accessible [18–22].

Another role for networks in the realm of the food insecurity is evident in research in urban settings, which indicates the importance of social networks in modulating whether or not individuals may express this condition [23]. Even in low-income households, there is some evidence that strong bonding social networks, may mitigate against other barriers, including cash income available for the purchase of food.

Globally, increasing awareness has be given to the relationship between agrobiodiversity and food security [24–26]. On occasion, households experiencing greater diversity are not in fact wealthier or necessarily more food secure [27]. Nevertheless, most research to date does affirm positive relationship between increased agricultural biodiversity and increased food security; with recent attention highlighting the necessity of such sources of variation in the face of climate change and the COVID-19 pandemic [28–30]. Additional work has also highlighted the role of food waste “valorization,” also through a network approach [31], which provides insight into the ‘output’ facets of exchange networks (that is, after food has been consumed).

### Justification for research

Our primary objective is to understand how the social position of households are related to diversity in agricultural production and trade in local food between households. We assess the potential consequences of environmental change on local production and the exchange of foodstuffs across community areas.

Specifically, we seek to better understand how both the diversity of food produced and collected by household members is related to the frequency and intensity of foodstuffs exchanged between households. Our efforts include the testing of accuracy in the reporting of production to determine these outcomes compared against the frequency and intensity of food exchanges between individual households. Finally, we are interested in how both the frequency and intensity of food exchanges may vary seasonally.

In keeping with these global and Amazonian-specific developments in research on food exchanges and agriculture, this paper focuses on the analysis of networks of exchanges of local food and their relationship to the agrobiodiversity of ribeirinho households in three geographically proximate communities in the Sustainable Development Reserve of Amanã. To better understand how the positions of households in these social networks of food exchanges, we posit that this relationship can be assessed through the diversity of agricultural production, quality and quantities of exchanges, and the season during which these exchanges most often take place. Methodologically, this research involves (1) mapping the social networks and affinities between households, (2) inventories of known species, and, finally, (3) statistical tests of the relationships between network and subsequent agrobiodiversity.

Agrobiodiversity should not be considered separate from the breadth of activities involved in the sustenance and yields derived from a cash economy. In fact, globally it has been shown that crop diversity may actually increase household cash incomes over time, especially in the face of uncertainty tied to monocultures and non-use of local varieties [32]. These are activities Amazonian riverine people (*ribeirinhos*) engage with daily. The role network brokers in SNA serve in these community dynamics is a topic of research and has not been investigated in ribeirinho communities in the area. Brokers are actors in social, economic, or political relations who facilitate access to valued resources [33]—in this case, households who facilitate access to valued food resources. Whether such “central” actors are both agriculturally productive as well as key to facilitating exchanges throughout communities under review via social networks is not fully understood. This articles seeks to explore whether cultivating and harvesting a wide diversity of produce may be related to community actors’ overall exchange behaviors.

### Setting

**Fig. 1:**
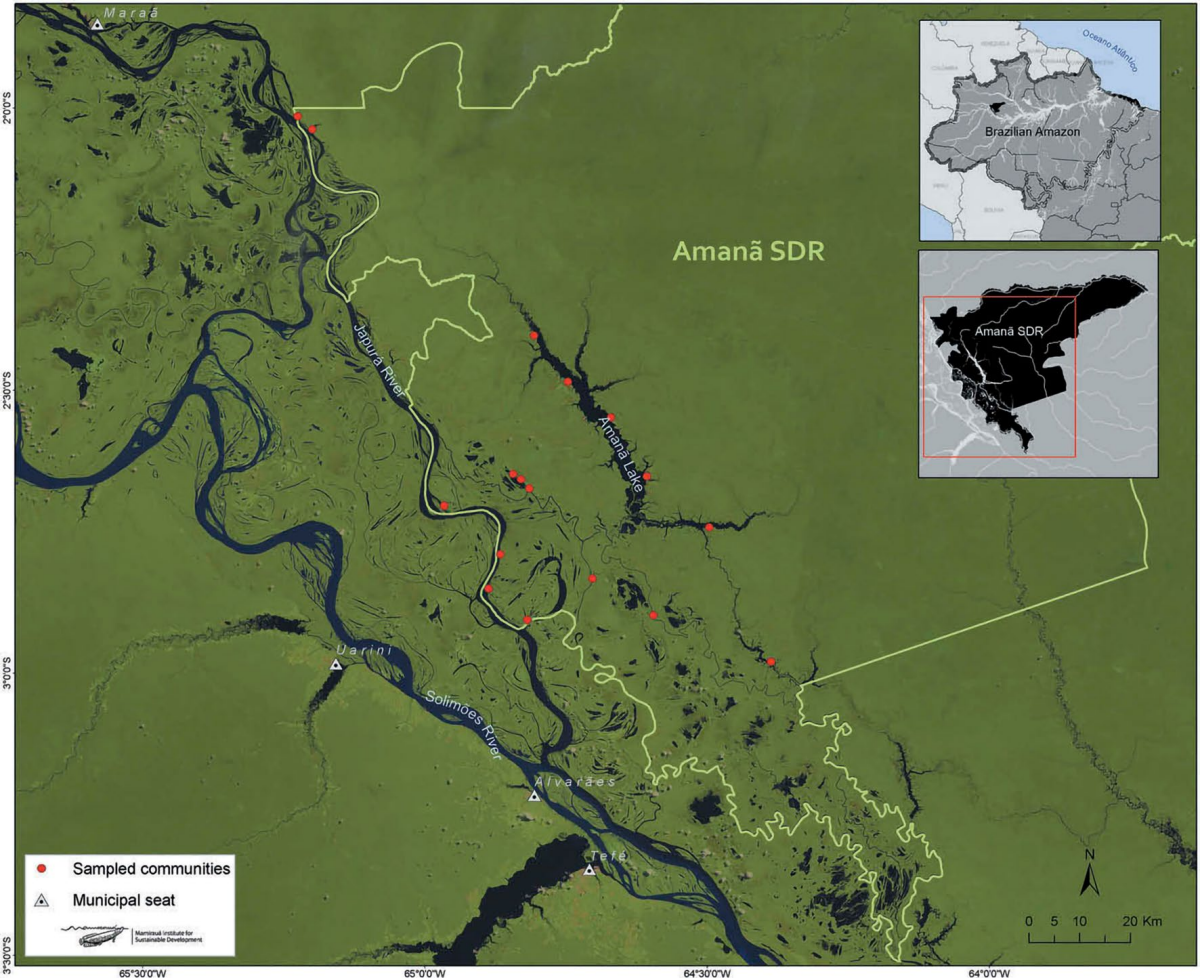
Location of communities in the study site

Research was conducted in the Amanã Sustainable Development Reserve (SDR), located in the Middle Solimões region (Fig. 1) A Sustainable Development Reserve (SDR) is a category within the Brazilian National System for Nature Conservation Units characterized as “a natural area inhabited by traditional peoples, whose life is based on sustainable use of natural resources, developed through generations and adapted to the local ecological conditions, performing the fundamental role of the protection of nature and maintenance of biological diversity.” Formed in 1996, it was the brainchild of researchers and residents who argued local populations were crucial in the monitoring of natural resources and conservation objectives for a diverse array of flora and fauna [34]. In 1998, the Amanã RDS was established between the Negro and Japurá Rivers at 01° 35′ S, 62° 44′ W and 03° 16′ S, 65° 23′ W. It covers an area of 2213 thousand hectares (approximately 5.5 million acres) of both upland and seasonally flooded areas, with an average annual water level fluctuation of 9–10 m. The Amanã RDS is overseen by the Mamirauá Institute for Sustainable Development (IDSM) and has a population of 3860 distributed throughout 80 villages or localities, composed of three or fewer households.

Two mediating factors should be taken into account when considering the research. Seasonality in tropical agriculture is in some respects less dramatic in tropical rather than temperate regions of the planet [35], but in várzea (flooded forest) ecosystems, is arguably as dramatic given the differences in mean water level and long periods when fields can be inundated [36, 37]. As such, the research was carried out over the course of 1 year (February 2014–February 2015), to account for intra-annual seasonality. In addition, as noted below, the personal network approach adopted for the project draws upon open-ended elicitation of other households (or alters), which in theory may lead to inaccurate responses [38]. However, due to the number of households in the communities as a whole (65), we de facto controlled for these conditions in the current model.

Rural Amazonian residents are frequently referred to in the academic literature as *caboclos* or *ribeirinhos*, terms describing mixed-race descendants of indigenous inhabitants of the Amazonian floodplains and European colonists. Researchers have pointed to a lack of recognition of these populations’ active entrepreneurial capabilities [39], as well as a dearth of research surrounding their agroecological knowledge [40].

Within the Amanã Reserve there exists a land tenure system unique to the Sustainable Development Reserve (RDS) model created in 1996, in which forms of private, alienable, and communal land rights exist. Transfers are enacted between members of the same community as prescribed by the RDS management plan and further elaborated by regional sectors (composed by community representatives) and the elected community heads themselves [41]. These practices must fall within the acceptable ranges for resource use, including extraction and land use modifications, as prescribed under management plans initiated by the RDS but enforced by state and federal agencies in full scope.

All three of the ribeirinho communities in this study—Nova Canaã, Matusalém, and São João do Ipecaçu—are located in the Amanã Sustainable Development Reserve and are within no more than 5 km of each other by boat (most of the year) or foot (the drying and dry seasons). While relatively diverse culturally and as pertains to kinship, such close-by settlements are undeniably linked. This reflects, in part, the socioeconomic and technological transformations, principally in transport, extent throughout the Brazilian Amazon since the early 2000s. While the most robust exchanges—a network’s density—occur within a given community, it’s also the case that from all the responses, as well as depicted in the graphs below, connectivity across components with geographic distance is decidedly present.

## Methodology

Over the course of more than 1 year, February 2014–February 2015, coinciding with the flooding and dry seasons in the Central Brazilian Amazon, 65 ribeirinho households were interviewed via questionnaires. Sixty-one of these households were interviewed four times throughout the year, during rainy, dry, flooding, and drying seasons (*cheia*, *vazante*, *seca*, *enchente*, respectively), while 5 were interviewed in three or fewer seasons. Between all the seasons under review, the “drying” (*vazante*) season, independent of any larger climate patterns, is historically understood to fall between March–May in the Central Amazon. As part of a larger survey conducted with researchers from Sustainable Development Reserve Institute of Mamirauá devoted to the administration of a census approach, respondents were asked to record their exchanges of food and during the most recent week of the response period. These questions consisted of dimensions related to type of foodstuff, frequency, and overall amount, as well as from and to whom exchanges were extracted.

**Fig. 2:**
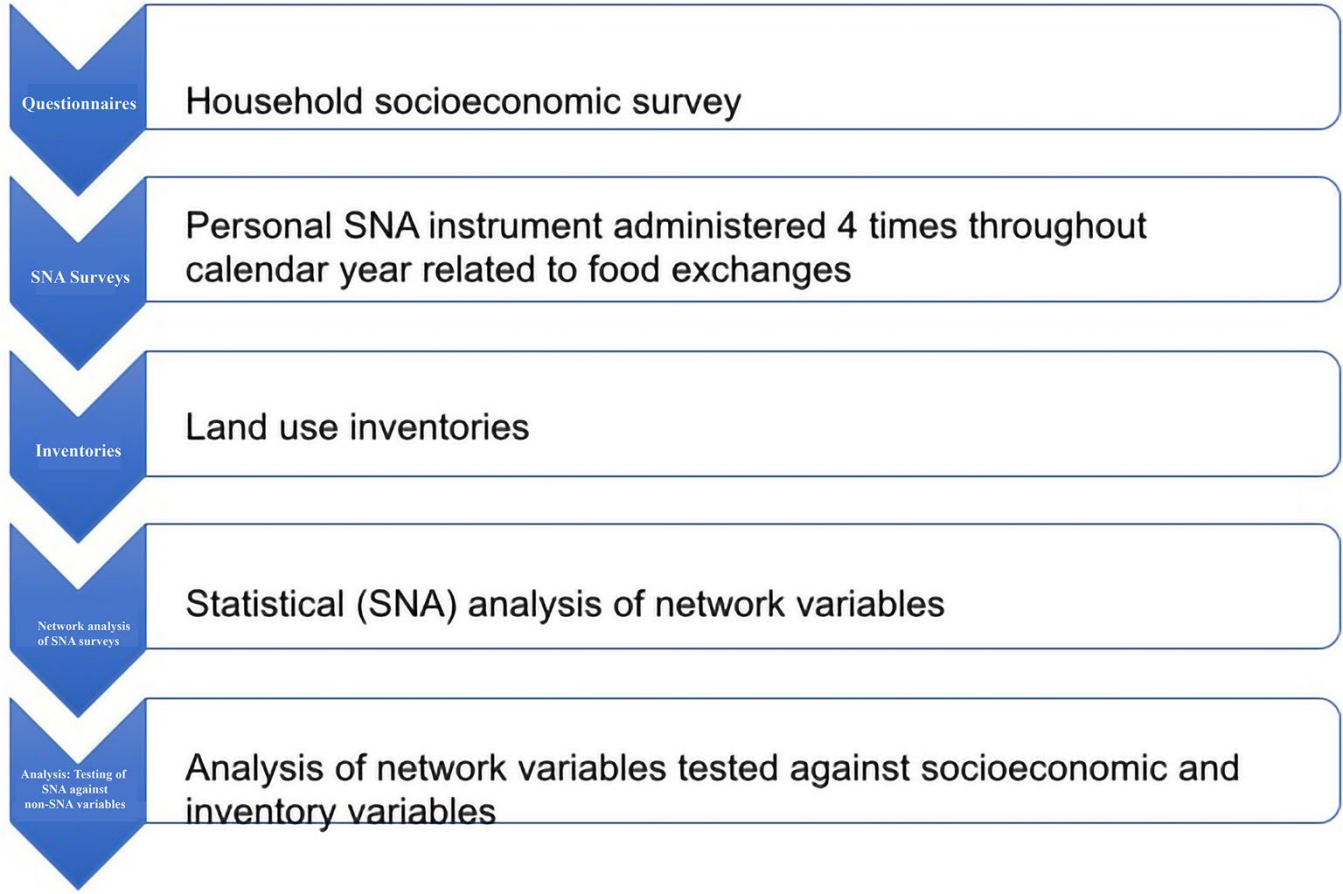
Research methodology utilized in this paper

Figure 2 illustrates the research methodology for this paper.

### Household questionnaires

Household income surveys were taken of each household in the study area between February 2014 and February 2015, following the instrument developed by the Sustainable Institute Mamirauá to measure wealth both directly and indirectly. The methodology for the survey design as it pertains to household income and a calculation of a score for it is obtainable from Peralta and Lima [42].

### Network surveys

To map the social networks of affinity, we conducted network interviews to measure the quantity and exchange of food over the last 7 days for each respective respondent. As in the case of the agrobiodiversity indices, four interviews per year were administered to adults, primarily but not exclusively male household members, linked to coincide with the four cycles of flooding.

Networks are formed by ties (edges) between actors (nodes) and are broadly categorized as either whole (sociocentric) or personal (egocentric) networks [43]. A sociocentric or whole network is a collection of data on relational ties within a group, such as a village or organization. Egocentric network data are collected from respondents (egos) who then provide information on their alters who could be family, friends or acquaintances. A personal network approach was utilized in this study given the understanding of the communities’ relative isolation (several hours by boat to the nearest urban center) and low population density (median population of study communities = 43 households).

We rely heavily on the use of centrality for most of this study (see Additional file 1 for a glossary of centrality terms). Centrality measures summarize structural position for a given node in a network [44, 45]. Degree, betweenness, and eigenvector centrality were all employed and are described in the analysis section below. Surveyed adults were randomly selected from within the household to reduce respondent bias [46]. The personal networks focus on the network interactions from the perspective of the respondent and have the advantage that they include network ties both within and outside the community in question. We gathered personal network data with survey questions about exchanges of food with other members of the three listed communities and some outside the study area depending on what was elicited from the personal network responses.

While conducting field research, any blatant tie discrepancies were subsequently resolved using a methodology described below. These explanations provided the equivalent of a qualitative context for a quantitative analysis. Since betweenness centrality requires reciprocated (symmetric) and binary (dichotomous) data, the data were symmetrized so that a report of net giving status was recoded as 1, any reports of equal exchange were marked by 0, and net receiver status was marked with a − 1. This symmetrization on the minimum ensures that the ties are reciprocated. Betweenness also requires connected networks; in disconnected networks, the betweenness scores are for betweenness within connected groups. The livelihood data were then added as attributes of each distinct household.

### Inventories

Prior research in the region has shown that the four commonly agreed upon land types for cultivation of edible and economic plants include *roça*, *sítios*, *quintais*, and *capoeiras* [47]. A roça is a swidden agricultural field that ranges in size between a few hundred square meters and occasionally more than a couple thousand and is marked primarily by the predominance of manioc (*Manihot esculenta*) and banana (*Musa* spp.). We counted up to 21 different landraces of manioc and 17 varieties of banana, but for the purposes of this research they were counted in the diversity index. While landrace and varietal diversity and richness can pose a compelling counter to a focus solely on species diversity [48, 49], such an approach was not adopted in this research. Depending on whether the roça is planted in an upland or lowland plot, it may also be marked by the presence of subsistence or variety crops intended to diversify nutrition and palates. A sítio is a semi-cultivated to heavily cultivated orchard, primarily focused on providing fruit—often for youth [14]. Quintais are home gardens, which may be the most species-, if not landrace-diverse of the land use types, but also represent the least coverage (and productivity) and are, therefore, not included for comparison purposes in this study. Finally, fallowing fields, also primarily swidden, between one season to a few years are referred to as capoeiras. They provide less intensively planted cultivars, ranging from pineapples to opportunistic food plants.

The households in the study were interviewed in relation to the food plant agrobiodiversity present in their lots, households, and other land types with repeated applications over the course of the entire year, spanning wet/dry and intermodal seasons. While not all households interviewed possessed extensive areas of manioc cultivation (*roça*), they did retain some combination of *quintal* (home garden), *capoeira* (fallow fields with loosely managed crops), and *sítio* (maintained forest lots or orchards). The interviews themselves took place initially in the household as a complement to other portions of the questionnaire referred to above, but subsequent field visits were also made to known household-tenured properties and those in abeyance, such as those with common-pool designations, where self-reported cultivation was indicated. Visits were conducted throughout the year for all the households assayed in this study.

We first identified species and varietal (landrace) diversity counts for each land type, where cultivation for food takes place. These quintais range between the smallest spatial area all the way to large roças. In between we also assayed less salubrious forested lots, sítios, and capoeiras. One step was a simple count of food crop species and landraces; in two of the communities, actual verification of the species and varietals was conducted. Both of these techniques would be considered in the framework of a bootstrap methodology [50, 51].

Second, we measured the standard deviation and then variance of species data. Efforts were made to standardize the interviews with whoever felt most confident discussing their food plant inventories during seasonal cycles in the same 1-week period. Occasionally, there were 1–2 weeks of difference between households interviewed, albeit all during one contiguous season.

## Results and discussion

### Production area’s profile

**Fig. 3:**
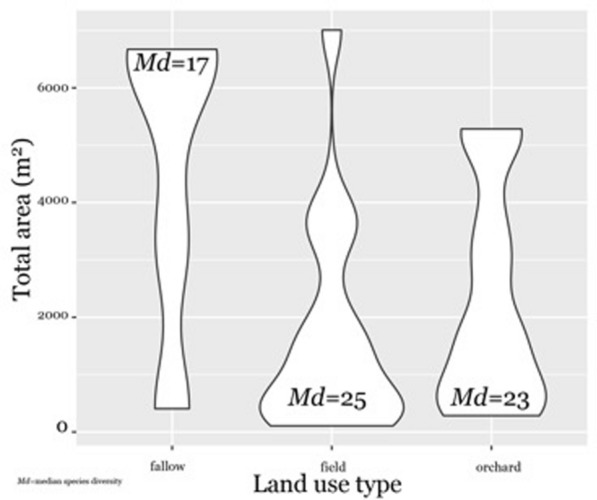
Violin plot of area (square meters) of land use types inset with median species diversity per unit

The diversity per area of each land type, where food cultivation or management takes place shows how home gardens, fields and orchards are areas of higher diversity and intense cultivation compared to fallow areas (Fig. 3). Quintais were not included given their high rates of diversity but small overall area and the presumption that species and landrace diversity would be higher (although not always richer). Given this, fields (*roças)* demonstrate the greatest median diversity but the lowest median total area (m^2^). Bananas were the most common food crop across all areas surveyed and manioc comes in second (Table 1), even considering the word “roça” is used interchangeably to refer to this staple crop. This demonstrates the significance of this crop in cultivation areas in general as well as its importance for food security (see Additional file 1 for all regression outputs).

**Table 1:** Frequency distribution graph of food plants cited in inventories across land use types in this research

Edible plant	Freq	%
*Musa* × *paradisiaca* (*banana*)	50	11.68539326
*Manihot esculenta* (*mandioca*)	46	10.6741573
*Euterpe oleracea* (*açaí*)	38	8.764044944
*Colocasia esculenta* (*cará*)	27	6.179775281
*Theobroma grandiflorum* (*cupuaçu*)	26	5.95505618
*Manihot esculenta* (*macaxeira*)	16	3.707865169
*Persea Americana* (*abacate*)	15	3.483146067
*Oenocarpus bacaba* (*bacaba*)	14	3.258426966
*Mangifera indica* (*manga*)	14	3.146067416
*Ananas comosus* (*abacaxi*)	13	2.921348315
*Bactris gasipaes* (*pupunha*)	13	2.921348315
*Bertholletia excelsa* (*castanha do Pará*)	12	2.808988764
*Citrus limon* (*limão*)	10	2.359550562
*Poraqueiba sericea* (*mari*)	10	2.359550562
*Pouteria caimito* (*abiu*)	9	2.02247191
*Astrocaryum aculeatum* (*tucumã*)	8	1.797752809
*Borojoa [Alibertia]* spp. (*apuruí*)	7	1.573033708
*Cucurbita pepo* (*girimum*)	6	1.460674157
*Saccharum officinarum* (*cana*)	6	1.348314607
*Inga edulis* (*ingá*)	6	1.348314607
*Syzygium jambos* (*jambo*)	6	1.348314607
*Mauritia flexuosa* (*buriti*)	5	1.235955056
*Cucurbita maxima* (*abóbora*)	5	1.123595506
*Capsicum chinense* Jaq. (*pimento cheirosa*)	5	1.123595506
*Theobroma cacao* (*cacau*)	4	1.011235955
*Capsicum annuum* L. (*pimento doce*)	4	1.011235955
*Syzygium cumini* L. (azeitona)	3	0.786516854
*Psidium guajava* (*goiaba*)	3	0.786516854
*Cucumis anguria* L. (*maxixe*)	3	0.786516854
*Zea mays* L. (*milho*)	3	0.786516854
*Carapa guianensis* Aubl. (*andiroba*)	3	0.674157303
*Averrhoa carambola* L. (*carambola*)	3	0.674157303
*Hevea brasiliensis* (*seringa*)	3	0.674157303
*Spondias mombin* (*taperibá*)	3	0.674157303
*Cocos nucifera* L. (*côco*)	2	0.561797753
*Citrus sinensis* (L.) Osbeck (*laranja*)	2	0.561797753
*Citrus* sp. (*lima*)	2	0.561797753
*Caryocar villosum* (Aubl.) Pers. (*piquiá*)	2	0.561797753
*Couma* sp. (*sorva*)	2	0.561797753
*Anacardium occidentale* L. (*caju*)	2	0.449438202
*Capsicum frutescens* L. (*pimento malagueta*)	2	0.449438202
*Psidium araca* (*araça*)	2	0.337078652
*Citrullus lanatus* (*melancia*)	2	0.337078652
*Malpighia glabra* L. (*acerola*)	2	0.224719101
*Oenocarpus mapora* (*bacabão*)	1	0.224719101
*Allium fistulosum* L. (*cebolinha*)	1	0.224719101
*Cedrela odorata* L. (*cedro*)	1	0.224719101
*Theobroma grandifloum* (*cubiu*)	1	0.224719101
*Theobroma subincanum* (*cupuí*)	1	0.224719101
*Bauhinia rutilans* (*arirão*)	1	0.112359551
*Platonia insignis* (*bacuri*)	1	0.112359551
*Rollinia deliciosa* (*biriba*)	1	0.112359551
*Myrciaria dubia* (*camu-camu*)	1	0.112359551
*Talinum fruticosum* (*caruru*)	1	0.112359551
*Eryngium foetidum* L. (*chicória*)	1	0.112359551
*Brassica oleracea* L. (*couve*)	1	0.112359551
*Artocarpus altilis* (*fruta-pão*)	1	0.112359551
*Annona muricata* L. (*graviola*)	1	0.112359551
*Artocarpus heterophyllus (jaca*)	1	0.112359551
*Cassia cowanii* (*mari-mari*)	1	0.112359551
*Endopleura uchi* (*uxi*)	1	0.112359551
*Capsicum baccatum* (*pimentão*)	1	0.112359551
*Citrus nobilis* Lour. (*tangerina*)	1	0.112359551
*Rheedia macrophylla* (*tartarugão*)	1	0.112359551
*Bixa orellana* (*urucum*)	1	0.112359551
Total	431	100

### Diversity and wealth

**Fig. 4:**
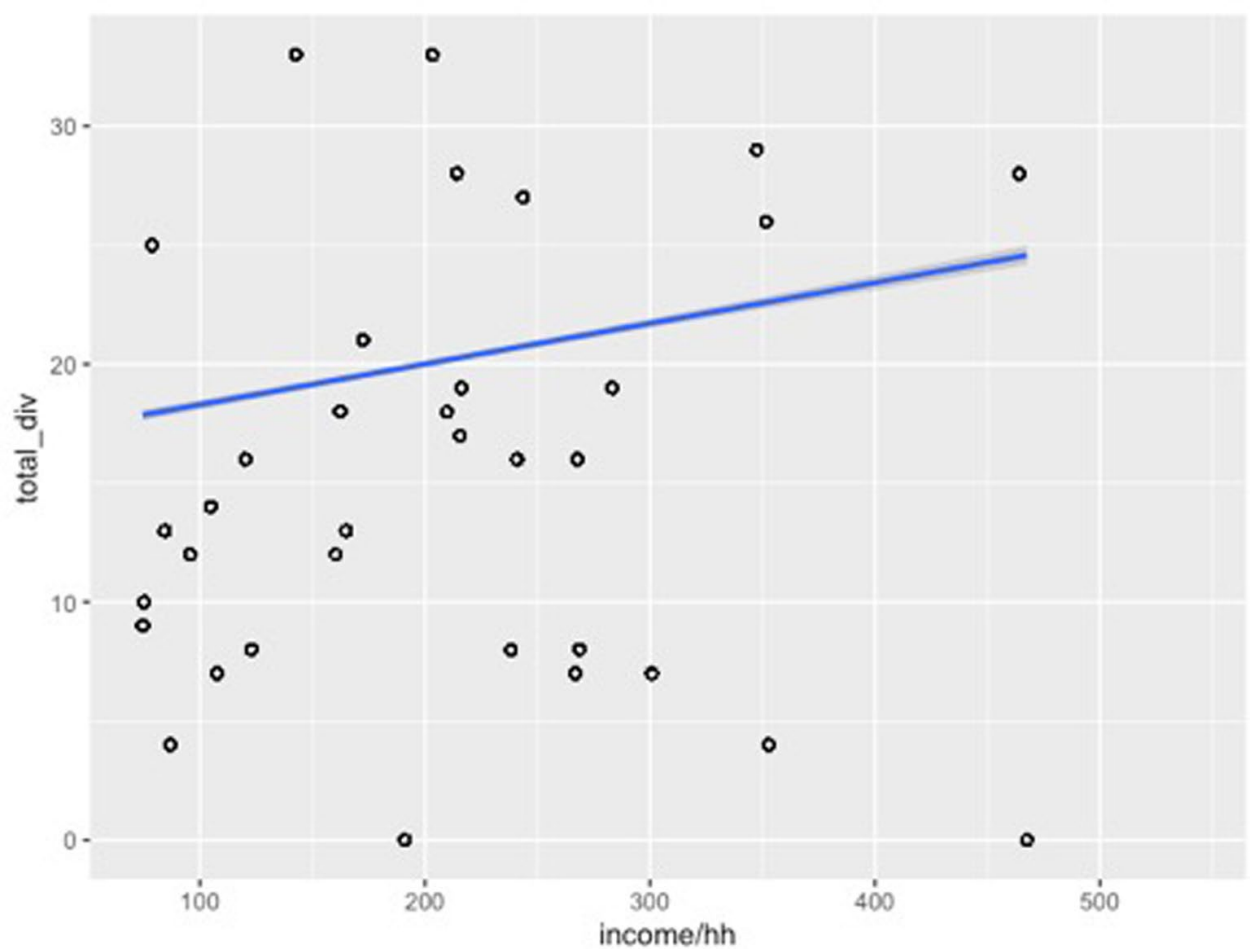
Relationship between income total diversity across all land use types. Our research demonstrates a slightly positive relationship between income and total agrobiodiversity

**Fig. 5:**
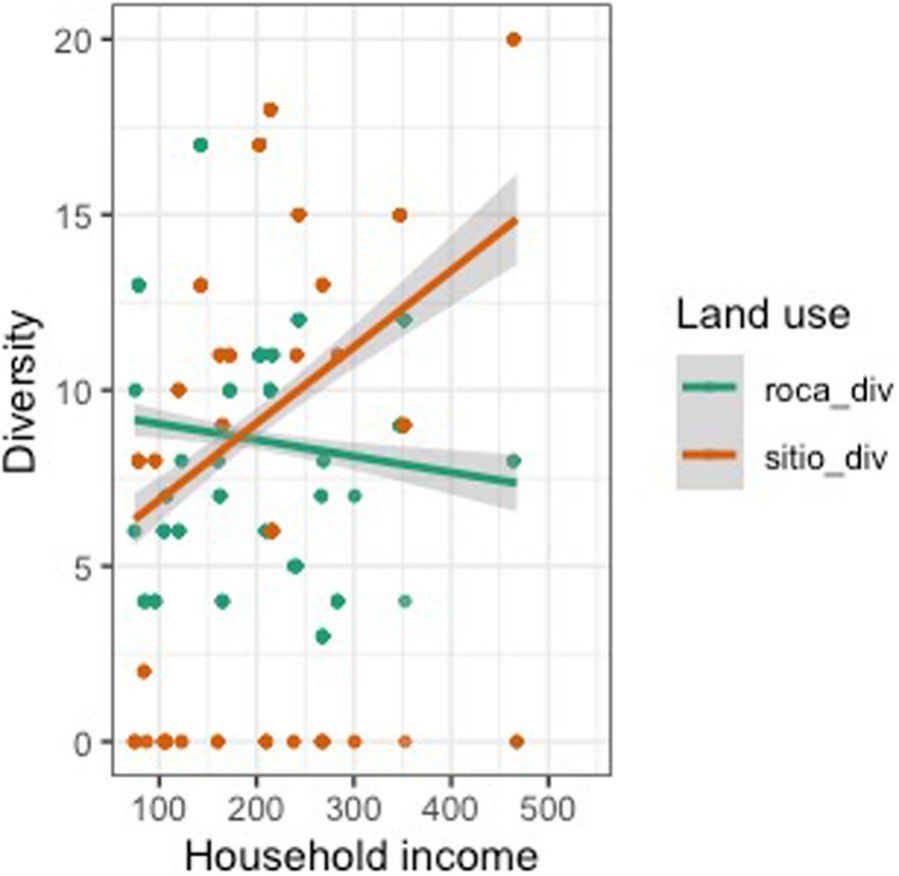
Comparison of agrobiodiversity plotted against income using only roças and sítios for analysis. Increased household income appears to predict greater sítio diversity, albeit with numerous households not in possession of a sítio. The inverse with roças

**Fig. 6:**
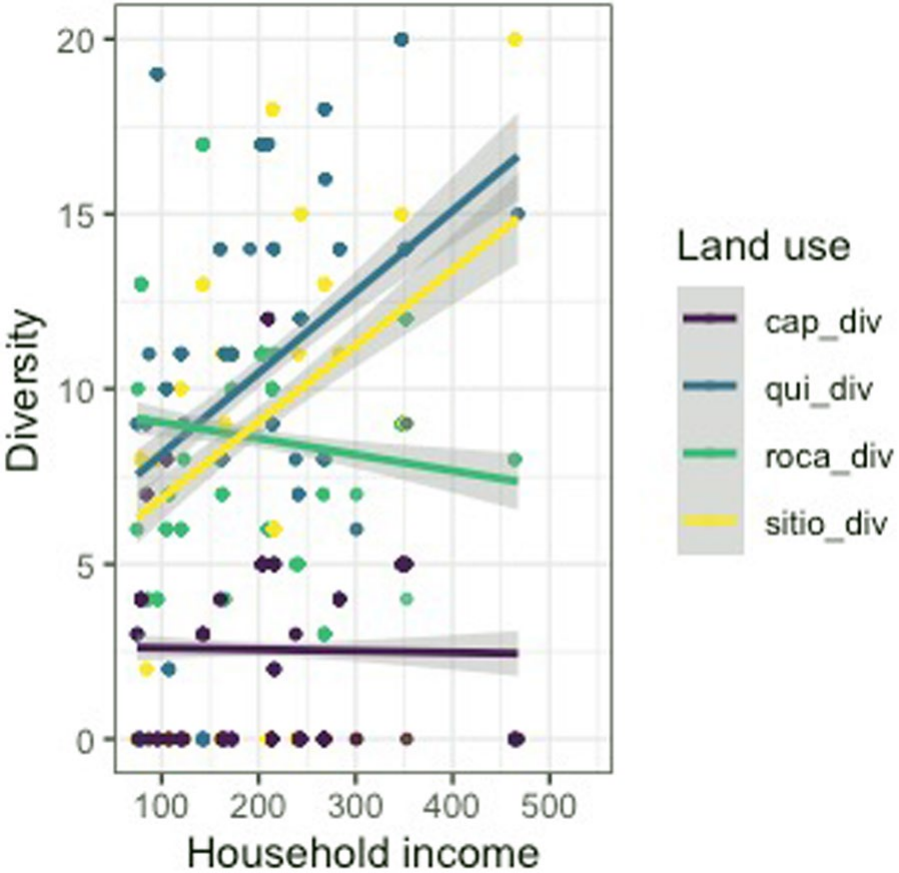
Plot of household income by land use diversity, in which all land use types are referenced. The majority of the households in the study did not utilize capoeiras as a land use practice

**Fig. 7:**
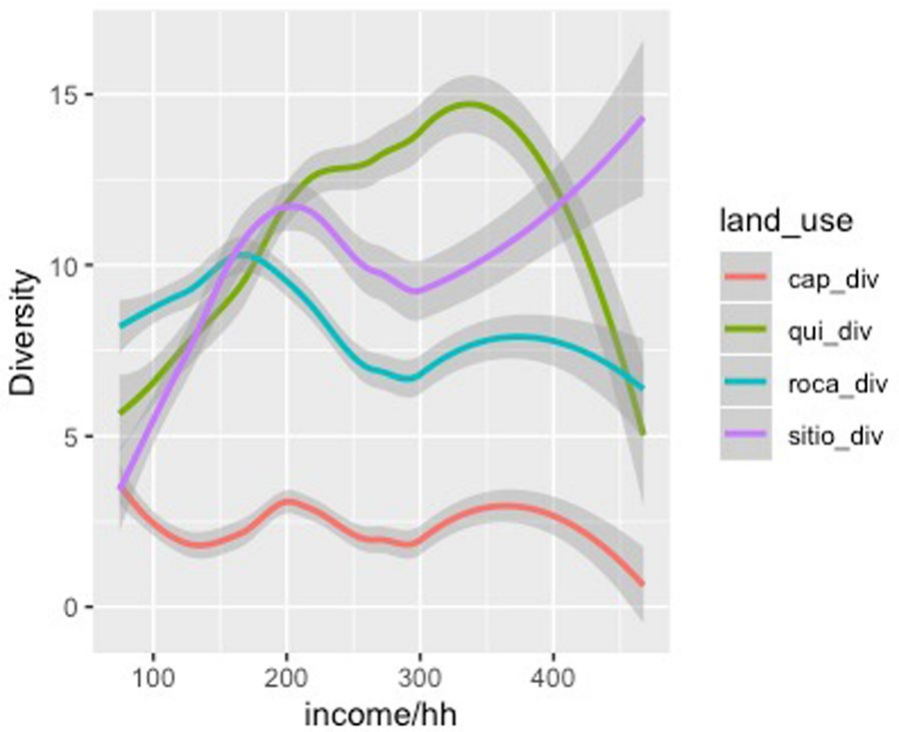
Line graph figure that depicts the relationship between household income and land use diversity. Income appears to predict progressively less agrobiodiversity in all land use types except for sítios, which appear to counter this trend

Our findings, however, indicate that a household’s income does appear to be strongly associated with the total agrobiodiversity across cultivation areas (Figs. 4, 5, 6 and 7). These results suggest that households with a higher income do value cultivation and management of agrobiodiversity as it pertains to food crops. When separated by land use, both sítio and quintal diversity appear to be the most directly related to income although in different respects, with sítios presenting increased diversity as household income increases and quintais reach a maximum before decreasing precipitously. Therefore, these areas could be viewed as adding diversity to diets, and perhaps labeled as peripheral areas. Conversely, agrobiodiversity in roças has a negative relationship to income, decreasing as income increases, suggesting these are areas, where households may rely upon food security given that staples, such as manioc and squash, as well as cash crops which of all the land use types is the most market-oriented (Additional file 1).

### Networks of food exchange

**Fig. 8:**
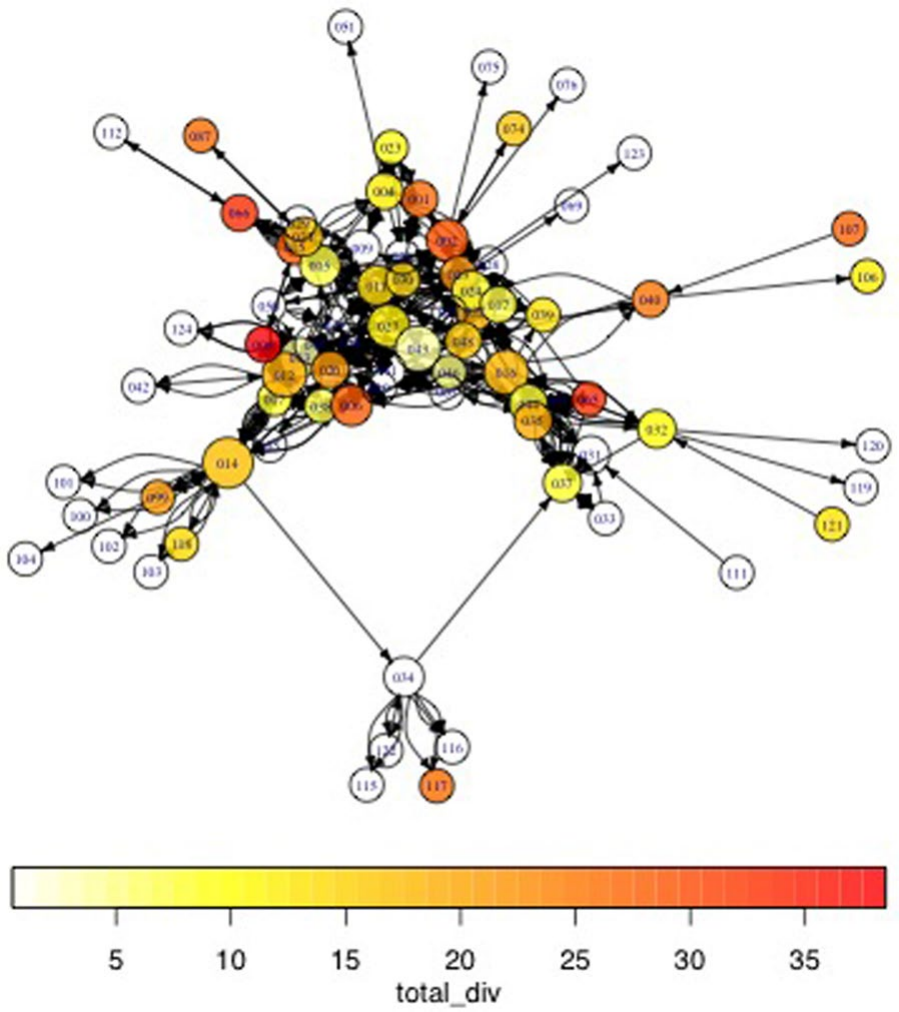
Total diversity of crops across land use types as measured against exchanges flooding, flooded, drying and dry seasons. The darker the color, the higher the household’s rate of diversity present. Larger nodes are more central households demonstrating high numbers of ties (both incoming and outgoing). Both statistically and visually, darker nodes tend to be larger—meaning that there is an apparent relationship between degree centrality and land use diversity across seasons. There are no marked differences in the subcomponents; however, there is one network clique that appears to tie households with minimal overall agrobiodiversity (node #034)

**Fig. 9:**
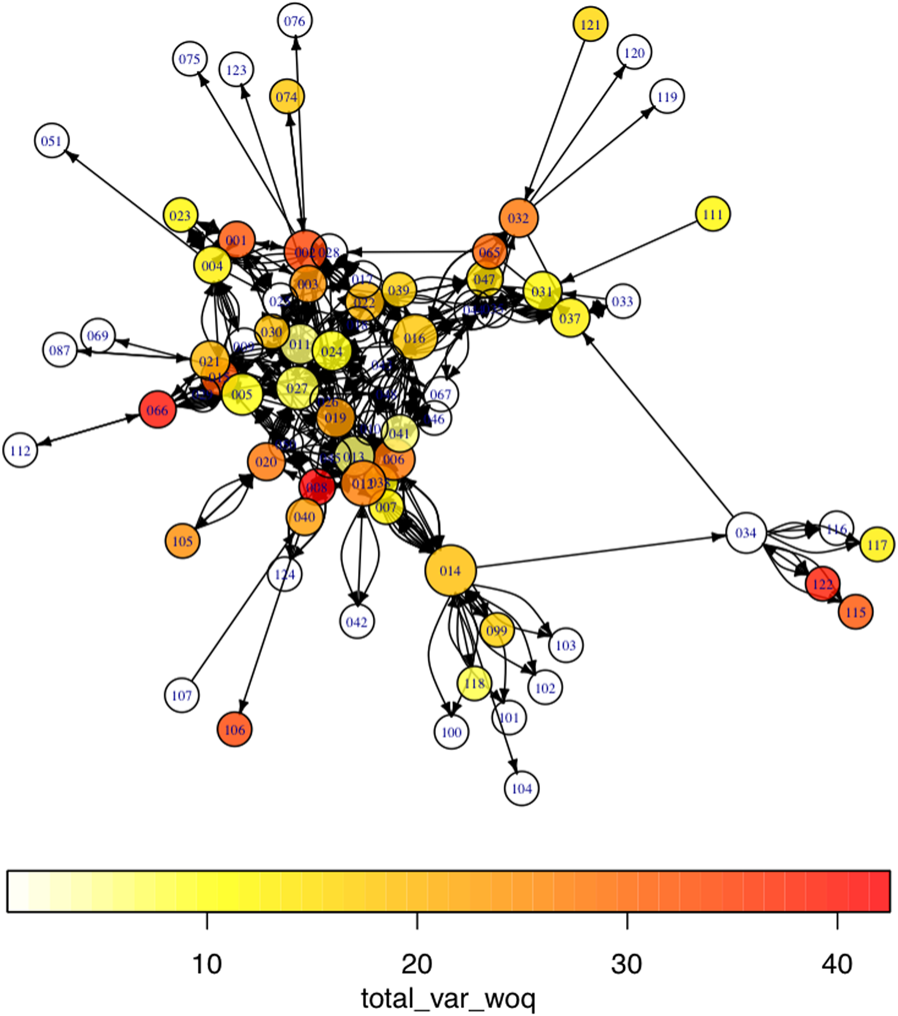
All season exchanges as measured utilizing the measurement for total diversity, save for yields from backyard gardens. The darker the color, the higher the rate of diversity evident. Larger nodes are more central in this graph, which reflects the statistical output. With some noticeable outliers, this graph demonstrates that a relationship exists between diversity (backyard gardens excluded) and centrality, in which the clique centered around 034 demonstrates greater diversity for it and related nodes

A household’s agrobiodiversity is significantly associated with the frequency and intensity of food exchanges between households. Moreover, networks of food exchanges from February 2014 to February 2015 have an observationally strong relationship to each other (Figs. 8 and 9). The lighter color circles (nodes) represent households across this span who cultivate a lower diversity of goods relative to their community peers. It is apparent to the eye that these households are less central to exchange networks, which is also borne out with network analysis statistics (their degree centrality values are lower). In other words, the higher a household’s observed diversity, the more frequent and intense exchanges between them are likely to be.

This runs counter to the argument for the existence of a so-called “economy of affection,” that is, the exchange of non-cash items amongst the Brazilian poor are not necessarily intended for bartering purposes (given that poorer households should arguably demonstrate less diversity and willingness to be left on their own. Therefore, their diet diversification does not appear to be linked to receiving foods from households demonstrating higher diversity.

Perhaps more remarkable is that the relationship between income and network measurements, wherein the most notable results are that with a higher income, a household’s indegree for food exchanges actually increases, and the inverse holds true for outdegree. That is, wealthier households received more fr0m exchanges and less wealthy households received fewer.

**Fig. 10:**
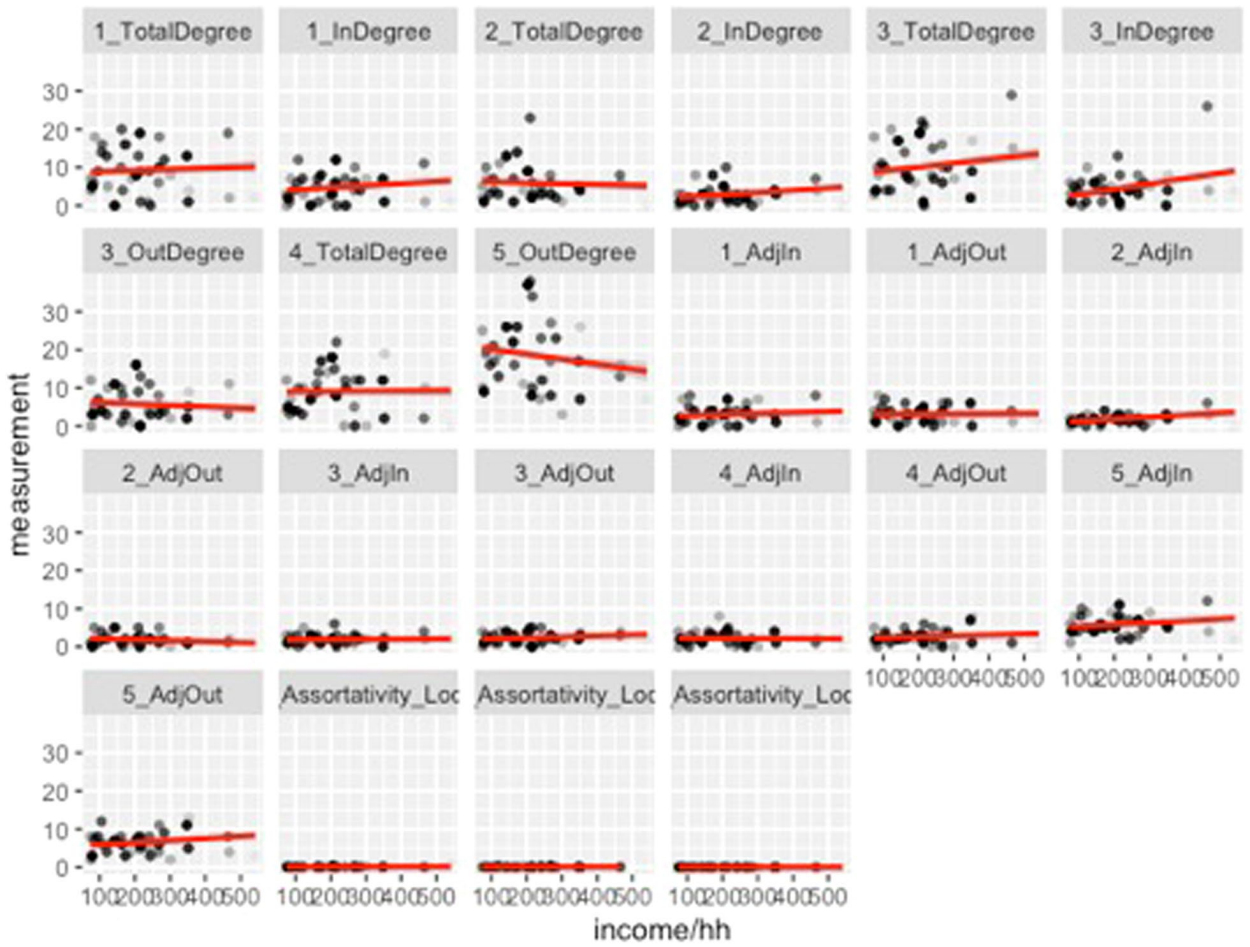
Scatterplots of household income and the statistically significant relationships with network measurements. Notably, outdegree (outbound tie) measurements appear to decreases as household incomes increase; indegree(inbound tie) measurements represent an inverse pattern. In simplified terms, these results demonstrate that households tend to give less in household exchanges as their incomes increase and provide more as their incomes decrease within a given study area

**Fig. 11:**
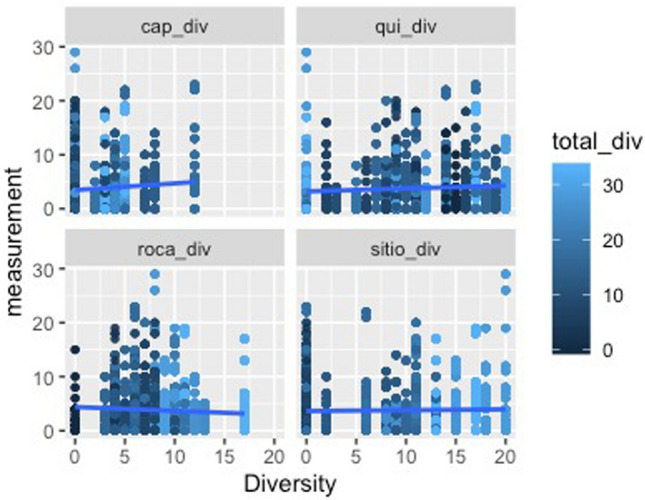
Diversity as measured against degree centrality, facetted by land use type. The total diversity of the households is expressed in color intensity. Of note, there is a negative relationship between roça diversity and degree centrality and a positive relationship between capoeira diversity and increased degree centrality. This is to say that the more agrobiodiverse a household’s roça is, the less central in a social network of food exchanges it tends to be, while a household that demonstrates greater agrobiodiversity in its sítios or capoeiras, as well as its quintais, the more central in a network of food exchanges it is

**Fig. 12:**
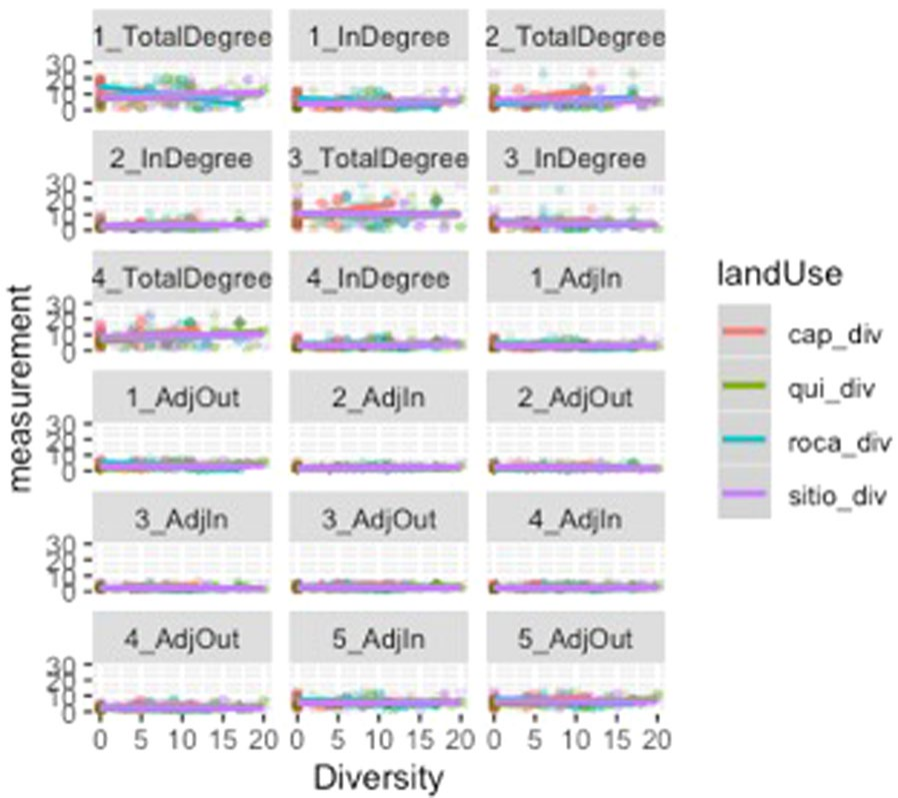
Scatterplots of the relationship between a household's agrobiodiversity and network measurements. Most visible are plots of the total degree or degree centrality against agrobiodiversity, as expressed in Fig. 5, but these measurements are depicted due to their statistically significant relationships as portrayed in the summaries of logistic regressions, which is used to model the probability of a certain event occurring. The most significant relationships are evident with roças and capoeiras. Notably, greater agrobiodiviersity seems to indicate less total and specifically indegree in a social network (inbound ties), while the opposite holds true for capoeiras. Outdegree was not found to be significant. Sítios and quintais also do not appear to show a dramatic relationship. Nevertheless, from the perspective of adjacency, which here would be defined as households that have numerous nearby ties (outbound or inbound), there is a telling relationship in that a household’s roça appears to be more agrobiodiverse as its role in the network figures more prominently; again, the opposite for capoeiras. Sítios and quintais remain stable in neither direction

Broken down by land type, some noteworthy features of the data appear. The most economically and food-important land use, as well as largest in spatial extent, roças demonstrate a negative relationship between degree centrality and diversity. This indicates that as a household demonstrates a more prominent role in a social network, its overall agrobiodiversity decreases significantly. *Capoeiras*, which are arguably the least valued land use given their designation as fallow fields, evince a positive relationship between degree centrality and diversity—in other words, a household with more agrobiodiversity in their capoeira is actually more central within a network of exchanges (the above visible in Figs. 10, 11 and 12).

Both utilizing multivariate and one-way analysis of variance, additional support for the association between agrobiodiversity and food exchange is visible. Empirically, the relationships between a household’s total diversity in their cultivated land and the position in a network of exchange across all three geographically proximate locations is of note. The differences in means between these samples is sufficient across all the assessed network measurements,^1^ with the least significance reported for total degree, factoring both incoming and outbound overall ties (Table 2).

**Table 2:** One-way multivariate analysis of variance (MANOVA) and analysis of variance (ANOVA) for variables across numerous network measurements

Tests using total diversity	Df	Pillai approx	*F* num	Df den	Df	Pr(> F)	Sum Sq	Mean Sq	*F* value	Residuals
MANOVA	1	0.61027	113.27	9	651	< 2.2e−16***				659
Out degree	1					0.000229***	199	198.87	13.69	863; 12,535; 14.52
Total degree	1					0.00391**	303	302.55	8.372	863; 31,188; 36.14
Assortativity local	1					< 2e−16***	1.303	1.3029	121.1	659; 7.089; 0.0108

Strongly significant relationships (the differences between means) are notable between total diversity and key markers of exchanges of food between households throughout a study area

Significance values: ***0, **0.001, *0.01

In contrast, the diversity of agricultural products farmers obtain from fallow field, capoeiras exclusively, is not linked to their sociometric roles in a network—that is, any meaningful statistical position within it. Capoeiras are a land use, where manioc, pineapple, banana and other opportunistic upland crops may be found throughout flooding seasons (Fig. 4). Capoeiras are also the least species- and landrace-rich of land uses in the region, and the flooding season Sep–Nov is one of the most challenging periods of the year to cultivate crops.^2^ The lack of a relationship may have much less to do with exchanges of specific crops from this land use type before they are weighted. Others are presented independently.

**Fig. 13:**
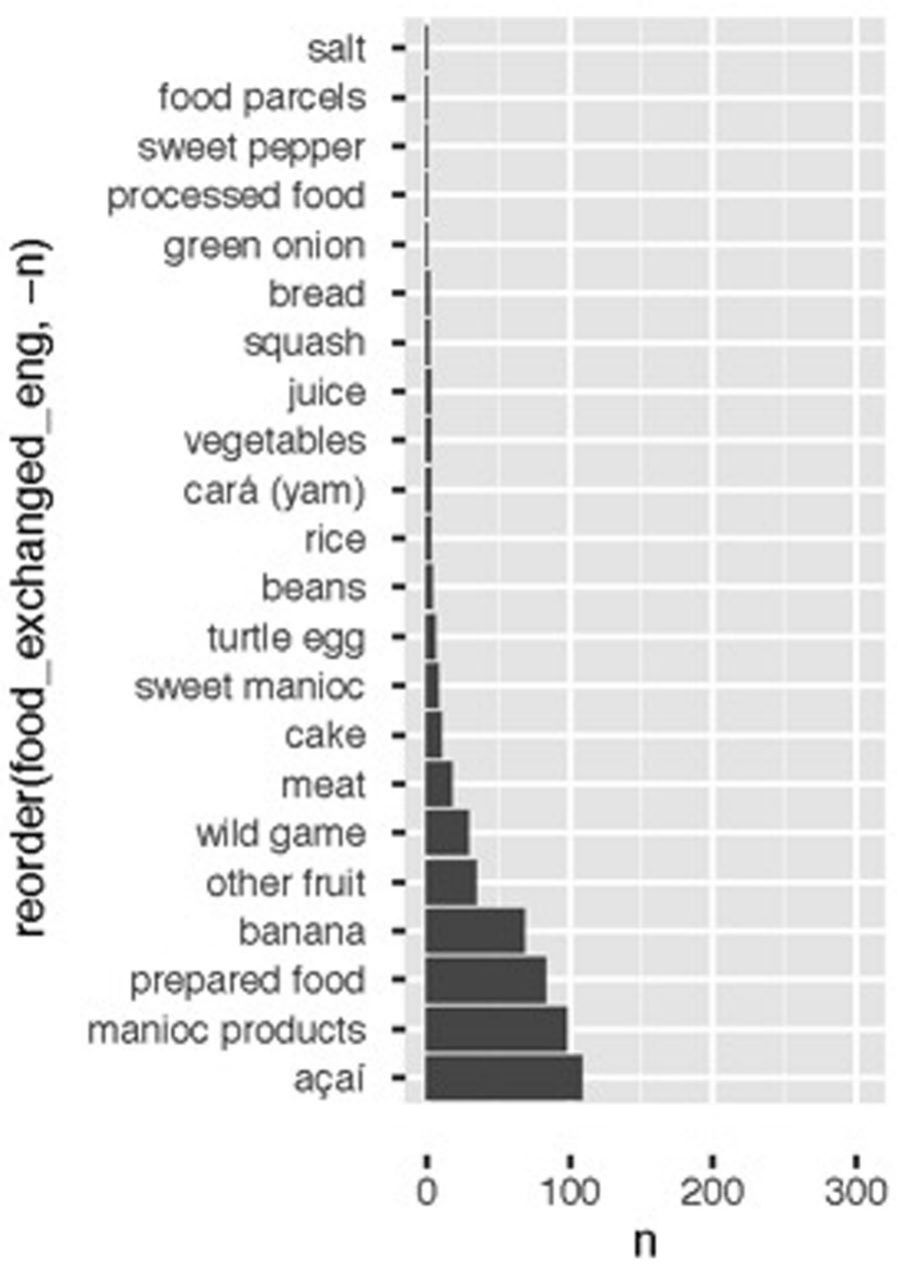
Exchanges across seasons and the foods most prevalent in exchanges. Açaí, a seasonal crop with some market value outside the communities. The third, prepared meals, were common forms of exchange across households

The types of food exchanged are demonstrative of the species and varietal diversity prevalent throughout the Amazon region, with perhaps the clearest example being fish inhabiting both the river as well as the pools and streams of the seasonally flooded forest (Fig. 13 is controlled for fish as it is far and away the most prevalent). Other non-agricultural foodstuffs also appear in heavy volume, including prepared food (*comida preparada*) and bushmeat (*carne do mato*). Overall, however, the majority of exchanged foods are derived from cultivated areas, either intensively or loosely. In addition, of these species, including banana and bitter manioc, the number of varietals number 20. Of note, a presence or absence method was utilized in this study.

Accuracy in reports of production corresponded with food exchange frequency and intensity assessed based on respondents’ reports about cultivated species alongside field observation in the field with residents and field assistants (ethno-botanical inventory) as seen in Table 3. Count data of these features from the survey responses and then the known data reveal that 4/67 possible households were cited in food exchange discrepancies (6%), of which 5 of a total 802 possible exchanges across all communities (0.62%). These discrepancies were related to fish and prepared food, not to any agricultural products per se (i.e., depending on the processing of the prepared dishes and to what degree their contents are primarily agricultural). Specific households in São João do Ipecaçu, the community, where all the discrepancies were identified and cited, appeared more than once across multiple seasons, indicating their reports were conflicting, including one household, 020A, where more than 12% of food exchanges were in opposition.

**Table 3:** Tie discrepancies for food exchanges of households in the study communities

Exchange ID	From	To	Direction	Community	Season	Food exchanged
63	045A	020A	1	SJI	1	Fish
32	020A	045A	1	SJI	1	Fish
33	020A	045A	1	SJI	1	Fish
14	019A	013A	− 1	SJI	3	Prepared food
11	013A	019A	− 1	SJI	3	Prepared food

These are households in which a reciprocal relationship was not identified (i.e., the respondent household [ego] identified an exchange [tie] with an alter, but the alter referenced did not confirm the tie)

045A = 0.048 accuracy (4.8%) * contested in that 020A claims there’s a connection that 045A does not and contested in terms of frequency and direction between a giver and a receiver in listed exchanges

020A = 0.125 (12.5%)

13A = 0.071 (7.1%)

19A = 0.043 (4.3%)

67 total HH: > 4/67 = reported differences: > 0.06 *in*accuracy among respondents (6%)

802 exchanges: > 5/802 = 0.006234413965 *in*accuracy for individual exchanges (0.63%)

**Fig. 14:**
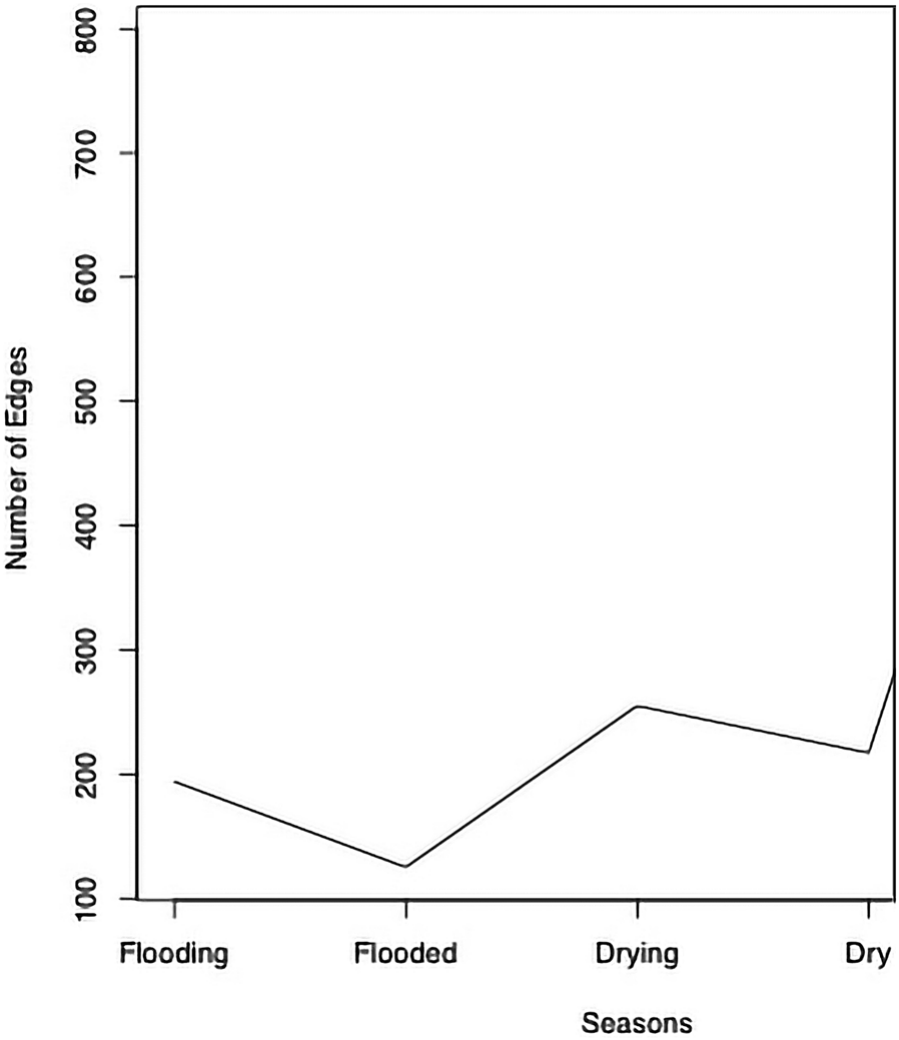
Graph demonstrating that the total number of ties, therefore, exchanges, are most prevalent during the “drying” (vazante) period, when waters recede but are not at their lowest point in the várzea flooded forest ecosystem (the dry [seca] season)

**Fig. 15:**
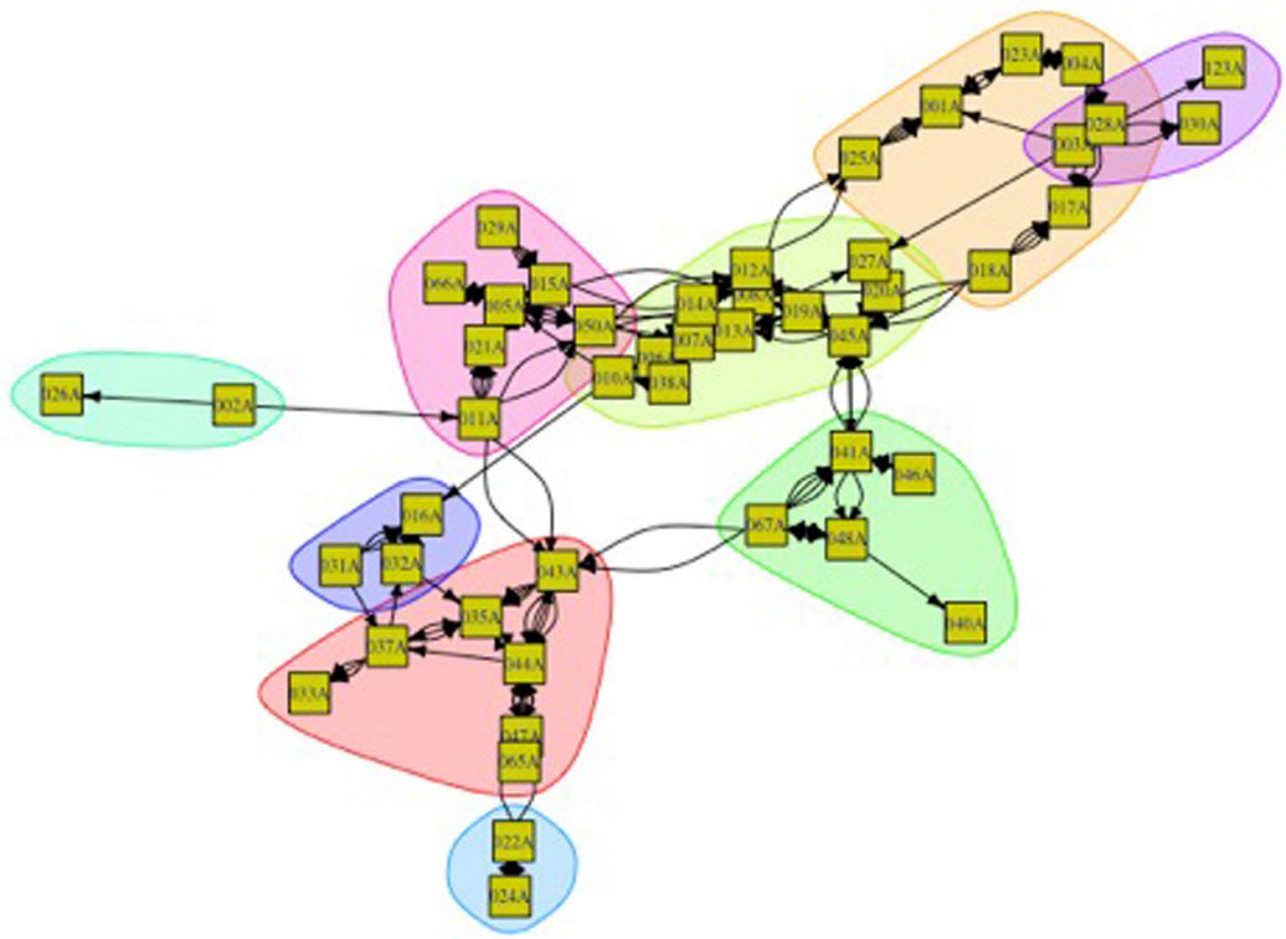
Dry season and subcomponents of exchanges between actors during the dry season. The densest cliques are residents of São João de Ipecaçu, the largest community in the study area

**Fig. 16:**
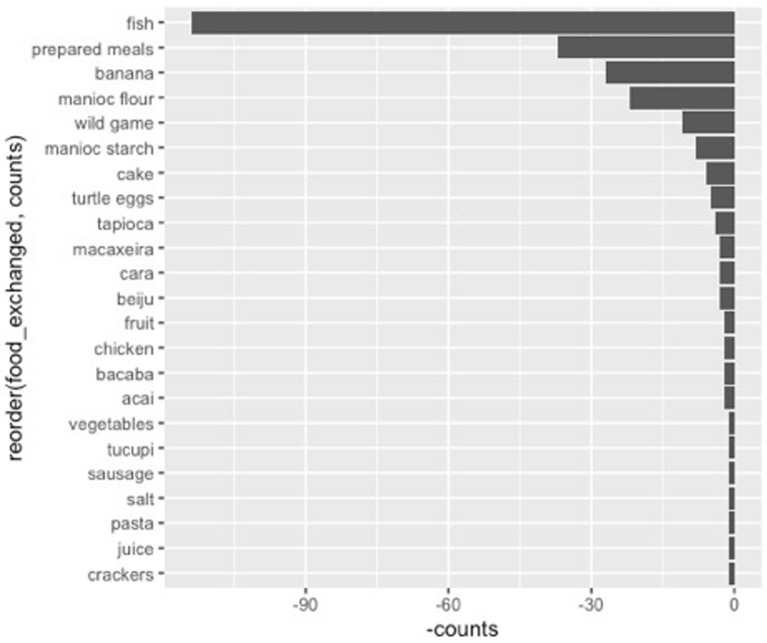
Food types exchanged across the drying (vazante) season, in which fish is even more prominent than during other periods, including the “dry” (seca) season, when significant fishing is carried out throughout the Amanã SDR, particularly in search of fish-like pirarucu (*Arapaima gigas*)

Whether the frequency and intensity of food exchanges vary seasonally is linked to the variability of foodways in rural Amazonia, and dependence on agricultural produce for exchanges is not exclusive. Network measurements across the study do vary by season as indicated by Wilcoxon signed rank tests to compare the location for each measurement yielding a *p* value of 0.125 or lower. The drying season is where the greatest number of food exchanges take place, and is statistically different from the other identified seasons in the region (Figs. 14, 15 and 16 and Table 4). During the this period, cliques are primarily formed around geographic boundaries across all communities, even though none are farther than 5 km apart (Fig. 1).

**Table 4:** Network-wide characteristics of resource sharing in the study communities of Amanã as varied by season

Season	Components	Diameter	Edges	Mutual_Edges	Asymmetric_Edges	Reciprocity	Assortativity_Global
Flooding	34	8	194	47	100	0.484536082	0.289411765
Flooded	33	11	125	25	75	0.4	0.251396648
Drying	31	14	255	57	141	0.447058824	0.215311005
Dry	15	11	217	45	127	0.414746544	0.135888502
All sesasons	1	8	791	220	351	0.556257901	0.267265193

Other studies in the area have shown that traditionally the drying may be one of the most abundant for certain fisheries, although not all [52–54], which may account for why fish appear prominently in the exchanges in this season relative to any specific designation of available agricultural goods.

## Discussion

A relationship persists between overall agrobiodiversity of the households in the study area and social position, notably in the case of betweenness and assortativity measurements. The former measures the role a node, in this case a household, acts as the broker between subcomponents of a network who might not otherwise be linked. Similarly, assortativity is a measure of the degree to which centrally located nodes connect to each other, in contrast to the manner households (nodes) possessing limited centrality connect to households that are less central in a network subcomponent. We show that there is an association between the diversity of species and landraces a household cultivates and their role as both providers and recipients in the network. Both indegree and outdegree measurements appear to have a relationship with the richness of what is garnered from the land—except for certain land types in less productive times of the year, the most statistically significant of these being the fallow field land use known as capoeira. Capoeiras are generally not managed with any intent to improve their overall productivity but may opportunistically include various crops.

Season is also important, but not exclusively for cultivation. As is well documented in much of the Central Amazon, fisheries play as much or more important role in terms of provision of calories and even economic yields for households along rivers and other waterways in the region, particularly during the dry season. Fish data was only collected at a general scale. It is marked that many of those households who serve in a brokering position across the networks exchange fish while also demonstrating a diverse array of crops and opportunistic edible plants in land use types ranging from home gardens to manioc fields solely a hectare in dimension.

## Conclusion

Agrobiodiversity cannot be considered separate from the breadth of activities focused on sustenance and yields from the cash economy, which rural people—including Brazilian *ribeirinhos*—engage in daily. It seems to be connected to quotidian social interactions and exchanges in both predictable and occasionally subtler ways. Those brokers who serve as prominent actors in rural riverine communities may not always be the most productive or in possession of the largest landholdings, although in some cases they are that as well, but their proclivity for cultivating and harvesting a wide diversity of produce may be equally important if not more so.

Agribiodiversity is largely measured in diverse ecosystems, such as the Central Brazilian Amazon, but throughout the Global South in the context of traditional populations and historical practices [55], particularly as employed by indigenous people. The depth of this diversity, however, also appears to be linked to exchanges and social interactions that are congruent with contemporary society. The ties of isolated communities are lower, but shares are made possible by modern forms of motorized transport [16], by the availability of industrialized fats and sugars necessary to produce and share foodstuffs considered luxuries a generation ago [14, 56], and communication technologies [57] allowing for up-to-date information on assorted subjects from the prices of manioc flour to the current environmental enforcement procedures vis-à-vis fisheries.

It has been hypothesized that agrobiodiversity is deeply connected to farmers’ abilities to navigate uncertainty at a subsistence level, and in some cases even profit in a cash economy, where monocropping or Green Revolution-era practices in the developing world were carried out. The results here may add nuance to this argument insofar that wealthier households (in terms of assets, cash and property) are actually recipients of food exchanges, while households that cultivate a wide diversity of crops are both active in granting and receiving exchanges of food relative to their less diverse peers. This inherently high level of activity in and out, as well as overall rates of agricultural productivity in farmers’ land uses, may lend a certain amount of agility and adaptability to ribeirinhos, and thus could indicate a reduction in the dependence on fossil fuels in their livelihood activities [58, 59], as nonrenewable energy inputs remain the hallmarks of many Green and post-Green Revolution agronomic projects in the Global South.

Future research may be well-advised to explore this possibility and how it helps shape discussions behind a different “green” revolution: the wholesale switch in investment away from nonrenewable sources and toward renewable ones [60]. This may occur in many forms of smallholder agriculture and less of its presence in social and economic activities thereafter, such as the exchange of food.

## Supplementary Information

**Additional file 1.** Glossary and regression model outputs. A glossary of centrality terms, as well as outputs for regression models used in the study.

## Data Availability

Anonymized data for this study is available on the Open Science Framework site.

## Footnotes

1 That is, the default range of measurements provided within the ‘igraph’ network package.

2 They may be, however, more species-diverse, although this research did not investigate this prospect.
