# Supplementary material for: Fruitful exchanges: social networks and food resources amidst change
Source: Agric Food Secur. 2022 Feb 16;11(1):15. doi: 10.1186/s40066-021-00342-5 (PMC8853342; doi:10.1186/s40066-021-00342-5)
Supplement: Supplementary file 1 — Additional file 1. Glossary and regression model outputs. A glossary of centrality terms, as well as outputs for regression models used in the study. [file 40066_2021_342_MOESM1_ESM.docx]

**APPENDIX**

**Glossary**

*Assortativity:* The assortativity coefficient is a measure of the amount of mixing between and across subgroups of individuals with certain attributes (sex, age, node degree) as compared to that expected by chance.

*Eigenvector:* An important node is connected to important neighbors. This is a measure of influence of a given node in the whole network. The notion is how well-connected a given node is with other well connected nodes in the network.

*Total degree:* Measures the number of connections with adjacent nodes.

If the network is directed, then a distinction is made between indegree and outdegree: indegree counts the number of ties directed to the node and outdegree is the number of ties that the node directs to others. Indegree means that many actors seek to direct ties to one actor. It may be regarded as a measure of importance of the actors receiving many ties. Actors who have high outdegree centrality may be relatively able to exchange with others, or disperse information quickly to many others. So actors with high outdegree centrality are often characterized as influential. Lots of ties coming in and lots of ties coming out of an actor would increase degree centrality. In the graph below, actor 3 has the highest degree centrality with six direct ties (1,2,4,6,7,9) and actor 1 is the next most central with four direct ties (2,3,8,9).

**Linear regression of income by total land use diversity**

Call:

lm(formula = `income/hh` ~ total_div)

Residuals:

Min 1Q Median 3Q Max

-135.77 -77.46 -26.43 70.54 300.55

Coefficients:

Estimate Std. Error t value Pr(>|t|)

(Intercept) 166.93251 1.81608 91.92 <2e-16 ***

total_div 1.90449 0.08283 22.99 <2e-16 ***

---

Signif. codes: 0 ‘***’ 0.001 ‘**’ 0.01 ‘*’ 0.05 ‘.’ 0.1 ‘ ’ 1

Residual standard error: 91.16 on 15728 degrees of freedom

(22 observations deleted due to missingness)

Multiple R-squared: 0.03252, Adjusted R-squared: 0.03246

F-statistic: 528.6 on 1 and 15728 DF, p-value: < 2.2e-16

**Multiple regression of diversity by network variables**

Call:

lm(formula = agrod5$total_div ~ ., data = agrod5[, c(1532:1581)])

Residuals:

Min 1Q Median 3Q Max

-2.626e-12 0.000e+00 0.000e+00 0.000e+00 3.290e-12

Coefficients: (28 not defined because of singularities)

Estimate Std. Error t value Pr(>|t|)

(Intercept) 3.063e+01 1.221e-12 2.508e+13 <2e-16 ***

`1_TotalDegree` -1.146e+01 3.154e-13 -3.632e+13 <2e-16 ***1532

`1_InDegree` 1.209e+01 5.688e-13 2.125e+13 <2e-16 ***1533

`1_OutDegree` NA NA NA NA

`2_TotalDegree` 6.126e-01 4.491e-14 1.364e+13 <2e-16 ***1535

`2_InDegree` 1.078e+01 1.025e-13 1.052e+14 <2e-16 ***1536

`2_OutDegree` NA NA NA NA

`3_TotalDegree` 2.303e+00 1.244e-13 1.850e+13 <2e-16 ***1538

`3_InDegree` 3.707e+00 8.261e-14 4.487e+13 <2e-16 ***1539

`3_OutDegree` NA NA NA NA

`4_TotalDegree` 4.760e+00 1.465e-13 3.248e+13 <2e-16 ***1541

`4_InDegree` -1.419e+00 7.966e-14 -1.782e+13 <2e-16 ***1542

`4_OutDegree` NA NA NA NA

`5_TotalDegree` NA NA NA NA

`5_InDegree` NA NA NA NA

`5_OutDegree` NA NA NA NA

`1_AdjIn` 1.905e+01 4.932e-13 3.863e+13 <2e-16 ***1547

`1_AdjOut` 6.179e+00 7.795e-14 7.926e+13 <2e-16 ***1548

`2_AdjIn` -1.962e+01 3.036e-13 -6.461e+13 <2e-16 ***1549

`2_AdjOut` -2.243e+01 7.567e-13 -2.965e+13 <2e-16 ***1550

`3_AdjIn` 3.325e+00 9.102e-13 3.653e+12 <2e-16 ***1551

`3_AdjOut` -1.571e+01 2.723e-13 -5.769e+13 <2e-16 ***1552

`4_AdjIn` 7.412e+00 3.237e-13 2.290e+13 <2e-16 ***1553

`4_AdjOut` -7.913e+00 6.247e-13 -1.267e+13 <2e-16 ***1554

`5_AdjIn` -2.146e+01 8.452e-13 -2.539e+13 <2e-16 ***1555

`5_AdjOut` 1.151e+01 4.738e-13 2.430e+13 <2e-16 ***1556

`1_Assortativity_Local` -1.891e+02 2.974e-12 -6.358e+13 <2e-16 ***1557

`2_Assortativity_Local` 1.388e+02 1.265e-12 1.098e+14 <2e-16 ***1558

`3_Assortativity_Local` -7.396e+02 2.271e-11 -3.257e+13 <2e-16 ***1559

`4_Assortativity_Local` 4.907e+02 1.304e-11 3.763e+13 <2e-16 ***1560

`5_Assortativity_Local` NA NA NA NA

`1_Closeness` NA NA NA NA

`2_Closeness` NA NA NA NA

`3_Closeness` NA NA NA NA

`4_Closeness` NA NA NA NA

`5_Closeness` NA NA NA NA

`1_Betweenness` NA NA NA NA

`2_Betweenness` NA NA NA NA

`3_Betweenness` NA NA NA NA

`4_Betweenness` NA NA NA NA

`5_Betweenness` NA NA NA NA

`1_EigenCentrality` NA NA NA NA

`2_EigenCentrality` NA NA NA NA

`3_EigenCentrality` NA NA NA NA

`4_EigenCentrality` NA NA NA NA

`5_EigenCentrality` NA NA NA NA

`1_Module` NA NA NA NA

`2_Module` NA NA NA NA

`3_Module` NA NA NA NA

`4_Module` NA NA NA NA

`5_Module` NA NA NA NA

---

Signif. codes: 0 ‘***’ 0.001 ‘**’ 0.01 ‘*’ 0.05 ‘.’ 0.1 ‘ ’ 1

Residual standard error: 3.211e-13 on 400 degrees of freedom

(293 observations deleted due to missingness)

Multiple R-squared: 1, Adjusted R-squared: 1

F-statistic: 1.532e+28 on 22 and 400 DF, p-value: < 2.2e-16

**Multiple regression of income by network variables**

Call:

lm(formula = agrod5$`income/hh` ~ ., data = agrod5[, c(1532:1581)])

Residuals:

Min 1Q Median 3Q Max

-8.773e-10 0.000e+00 0.000e+00 0.000e+00 1.840e-10

Coefficients: (28 not defined because of singularities)

Estimate Std. Error t value Pr(>|t|)

(Intercept) 1.839e+03 1.809e-10 1.016e+13 <2e-16 ***

`1_TotalDegree` -4.303e+02 4.673e-11 -9.209e+12 <2e-16 *** 1532

`1_InDegree` 8.475e+02 8.428e-11 1.006e+13 <2e-16 *** 1533

`1_OutDegree` NA NA NA NA

`2_TotalDegree` -8.465e+01 6.653e-12 -1.272e+13 <2e-16 *** 1535

`2_InDegree` 3.450e+01 1.519e-11 2.271e+12 <2e-16 ***1536

`2_OutDegree` NA NA NA NA

`3_TotalDegree` -2.571e+02 1.844e-11 -1.394e+13 <2e-16 ***1538

`3_InDegree` 1.812e+02 1.224e-11 1.480e+13 <2e-16 ***1539

`3_OutDegree` NA NA NA NA

`4_TotalDegree` 2.637e+02 2.171e-11 1.215e+13 <2e-16 ***1540

`4_InDegree` -3.829e+01 1.180e-11 -3.245e+12 <2e-16 ***1541

`4_OutDegree` NA NA NA NA

`5_TotalDegree` NA NA NA NA

`5_InDegree` NA NA NA NA

`5_OutDegree` NA NA NA NA

`1_AdjIn` 5.677e+02 7.308e-11 7.769e+12 <2e-16 *** 1546

`1_AdjOut` -5.497e+01 1.155e-11 -4.760e+12 <2e-16 *** 1547

`2_AdjIn` 1.088e+02 4.498e-11 2.418e+12 <2e-16 *** 1548

`2_AdjOut` -9.824e+02 1.121e-10 -8.763e+12 <2e-16 *** 1549

`3_AdjIn` 1.309e+03 1.349e-10 9.707e+12 <2e-16 *** 1550

`3_AdjOut` 1.747e+02 4.035e-11 4.329e+12 <2e-16 *** 1551

`4_AdjIn` 4.731e+02 4.795e-11 9.866e+12 <2e-16 *** 1552

`4_AdjOut` -1.099e+03 9.255e-11 -1.187e+13 <2e-16 *** 1553

`5_AdjIn` -1.203e+03 1.252e-10 -9.609e+12 <2e-16 *** 1554

`5_AdjOut` 6.925e+02 7.020e-11 9.866e+12 <2e-16 *** 1555

`1_Assortativity_Local` 6.249e+01 4.406e-10 1.418e+11 <2e-16 *** 1556

`2_Assortativity_Local` 4.609e+02 1.874e-10 2.460e+12 <2e-16 *** 1557

`3_Assortativity_Local` -3.136e+04 3.364e-09 -9.324e+12 <2e-16 *** 1558

`4_Assortativity_Local` 1.589e+04 1.932e-09 8.225e+12 <2e-16 *** 1559

`5_Assortativity_Local` NA NA NA NA

`1_Closeness` NA NA NA NA

`2_Closeness` NA NA NA NA

`3_Closeness` NA NA NA NA

`4_Closeness` NA NA NA NA

`5_Closeness` NA NA NA NA

`1_Betweenness` NA NA NA NA

`2_Betweenness` NA NA NA NA

`3_Betweenness` NA NA NA NA

`4_Betweenness` NA NA NA NA

`5_Betweenness` NA NA NA NA

`1_EigenCentrality` NA NA NA NA

`2_EigenCentrality` NA NA NA NA

`3_EigenCentrality` NA NA NA NA

`4_EigenCentrality` NA NA NA NA

`5_EigenCentrality` NA NA NA NA

`1_Module` NA NA NA NA

`2_Module` NA NA NA NA

`3_Module` NA NA NA NA

`4_Module` NA NA NA NA

`5_Module` NA NA NA NA

---

Signif. codes: 0 ‘***’ 0.001 ‘**’ 0.01 ‘*’ 0.05 ‘.’ 0.1 ‘ ’ 1

Residual standard error: 4.757e-11 on 400 degrees of freedom

(293 observations deleted due to missingness)

Multiple R-squared: 1, Adjusted R-squared: 1

F-statistic: 8.621e+25 on 22 and 400 DF, p-value: < 2.2e-16
